# Areas Recruited during Action Understanding Are Not Modulated by Auditory or Sign Language Experience

**DOI:** 10.3389/fnhum.2016.00094

**Published:** 2016-03-08

**Authors:** Yuxing Fang, Quanjing Chen, Angelika Lingnau, Zaizhu Han, Yanchao Bi

**Affiliations:** ^1^State Key Laboratory of Cognitive Neuroscience and Learning and IDG/McGovern Institute for Brain Research, Beijing Normal UniversityBeijing, China; ^2^Center for Mind/Brain Sciences, University of TrentoRovereto, Italy; ^3^Department of Psychology and Cognitive Science, University of TrentoRovereto, Italy; ^4^Department of Psychology, Royal Holloway University of LondonEgham, UK

**Keywords:** action recognition, mirror neuron, deaf, auditory experience, sign language

## Abstract

The observation of other people’s actions recruits a network of areas including the inferior frontal gyrus (IFG), the inferior parietal lobule (IPL), and posterior middle temporal gyrus (pMTG). These regions have been shown to be activated through both visual and auditory inputs. Intriguingly, previous studies found no engagement of IFG and IPL for deaf participants during non-linguistic action observation, leading to the proposal that auditory experience or sign language usage might shape the functionality of these areas. To understand which variables induce plastic changes in areas recruited during the processing of other people’s actions, we examined the effects of tasks (action understanding and passive viewing) and effectors (arm actions vs. leg actions), as well as sign language experience in a group of 12 congenitally deaf signers and 13 hearing participants. In Experiment 1, we found a stronger activation during an action recognition task in comparison to a low-level visual control task in IFG, IPL and pMTG in both deaf signers and hearing individuals, but no effect of auditory or sign language experience. In Experiment 2, we replicated the results of the first experiment using a passive viewing task. Together, our results provide robust evidence demonstrating that the response obtained in IFG, IPL, and pMTG during action recognition and passive viewing is not affected by auditory or sign language experience, adding further support for the supra-modal nature of these regions.

## Introduction

Action understanding supports the interpretation of others’ goals, intentions, and reasons (Brass et al., 2007). We can understand actions presented from both visual and auditory inputs, with potential interactions between the two modalities. Behaviorally, Thomas and Shiffrar (2010) found that detection sensitivity improved when point-light displays of human actions were paired with veridical auditory cues (footsteps) but not when paired with simple tones. On a neural level, the human mirror system (hMS), consisting of the posterior inferior frontal gyrus (IFG), and the inferior parietal lobule (IPL), and the superior temporal sulcus (STS) have been consistently suggested to play a crucial role in action understanding (Iacoboni et al., 1999; Rizzolatti et al., 2001; Carr et al., 2003; Buccino et al., 2004; Rizzolatti and Craighero, 2004; De Lange et al., 2008; Caspers et al., 2010; Caramazza et al., 2014). The macaque mirror neuron system shows multisensory properties: neurons in area F5 fire when an action is performed, heard, or seen (Kohler et al., 2002; Keysers et al., 2003). Likewise, the hMS has been reported to be activated not only when observing actions but also when listening to action-related sounds (Lewis et al., 2005; Gazzola et al., 2006; Lahav et al., 2007).

To understand the manners in which hMS activation reflects processes of modality-specific visual or auditory properties of actions, a set of elegant studies examined brain activation obtained during action observation in special populations that are deprived of visual or auditory experiences, i.e., blind or deaf individuals. A puzzling picture emerged, however. While hMS activation during action observation in congenitally blind participants was observed to be largely similar (Ricciardi et al., 2009) or partly similar (in IFG; Lewis et al., 2011) to hearing controls, two studies have reported that congenital deaf individuals do not engage the IPL and IFG, the regions they defined to be hMS, during action observation. Corina et al. (2007) used PET to study deaf signers and controls during passive observation of self-oriented actions, object-oriented actions, and actions used in American Sign Language (ASL). They found that the left frontal and posterior superior temporal language areas were activated during the observations of ASL actions, whereas the two non-linguistic action types elicited activation in middle occipital temporal-ventral regions, but not the hMS. By contrast, hearing individuals exhibited a robust activation in the hMS for all three types of action. Emmorey et al. (2007) reported that passive viewing of pantomime actions and action verbs used in ASL yielded little activation in the hMS in deaf signers. Despite the inconsistent results regarding the ASL condition, both studies suggested that deprivation of auditory experience and/or gaining of ASL experience modulates the activation of the hMS, at least the IFG and IPL regions.

These findings pose an intriguing question to the mechanisms underlying the activation of the hMS. Why does visual experience, presumably the dominant modality through which action is perceived and understood, not appear to affect the recruitment of the hMS during action observation, whereas auditory experience seems to have an effect? The interpretation being proposed (Emmorey et al., 2010) was that besides the deprivation of auditory experience, the deaf population also differs from the controls in having a different modality of linguistic experience, i.e., sign language, which heavily relies on action observation and understanding. The possibility that language experience may modulate the activation of the hMS was raised by previous studies where deaf signers exhibited no hMS activation during action observation. It was suggested that the extensive training with comprehending sign language, which is expressed by sophisticated hand/upper body actions, deaf signers might be more efficient and automatic than controls, at least when the action tasks do not explicitly require action understanding (e.g., passive viewing). It is thus empirically open whether these regions would be recruited to support action understanding (rather than passive viewing) in congenitally deaf signers similarly to hearing controls without sign language experience.

To examine whether deaf signers’ neural activity in processing actions is different to hearing controls only during passive action observation tasks, we employed tasks where explicit responses based on action understanding are required. We further tested the manner in which the tentative plasticity in deaf was attributable to sign language experience by examining whether the hMS responses in the deaf are modulated by the nature of the actions (sharing similar effectors with sign language or not) and sign language experience. If the functionality of hMS in hearing controls is only altered in deaf signers when action understanding is required, we would predict similar hMS activation for deaf and hearing participants (Experiment 1). Furthermore, if the tentative plasticity is driven by sign language usage, we expect greater plastic changes for arm actions (common effector with sign language actions) relative to leg actions, and for deaf individuals with longer sign language experience.

Specifically, in Experiment 1 the level of processing required for action processing (goal/effector vs. low level perception) and the type of effector (arm, leg) were manipulated. Point light animations, which were used to encourage action understanding with minimal properties of non-motion aspects, depicted either arm- or leg-related actions. Participants had to either detect whether one of the dots briefly turned red (Red Dot Task), judge whether the action consisted of a movement of the arm or leg (Effector Task) or judge the goal of the action (Goal Task; Lingnau and Petris, 2013). The Red Dot Task was specifically designed to control for the “low level” stimuli properties, allowing to more specifically tap into the process engaged during understanding an action. A group of deaf participants with varying degrees of sign language experience were enrolled.

In addition to Experiment 1, we carried out Experiment 2 to replicate findings of previous passive viewing studies of deaf signers (Corina et al., 2007), using human action videos similar to those used by Corina et al. (2007). The two experiments were carried out in one scanning session, but differed with respect to the underlying designs and rationales, and are thus reported separately below.

## Experiment 1: Judgment of Point-Light Display Actions

### Methods

#### Participants

Thirteen congenitally deaf individuals (two males) and 13 hearing individuals (two males) participated in Experiment 1. One deaf individual was discarded during data analysis due to excessive head motion. One run of a hearing participant was discarded due to an unexpected pause of the scanner. All participants were undergraduate students, with deaf participant recruited from a department specialized for disable students in a local community colledge and the hearing control group from Beijing Normal University, with normal or corrected-to-normal vision, with no history of any neurological disorders, were right-handed (Edinburgh Handedness Questionnaire) except for one deaf subject who was ambidextrous. All deaf individuals (mean age = 20.4 years; *SD* = 1.45; range: 17–22 years) reported a profound hearing loss (≥95 dB), were using Chinese Sign Language (CSL, mean acquired age = 6.5 years; *SD* = 3.2; range: 2–12), and had poor articulation intelligibility. To test the ability of the articulation intelligibility, 11 deaf subjects were asked to read aloud 30 words. Two naive raters judged the speech intelligibility on a 5-point scale (0 – 4, 4 being most clear), with high inter-rater reliability (Spearman’s ρ = 0.94). The mean rating was 0.8 (range: 0.1–2.8, *SD*: 0.73). More information about the written and phonological ability of this deaf participants could be found in Wang et al. (2015). The hearing participants (mean age = 20.5 years; *SD* = 1.13; range: 19–23 years) reported normal hearing and no knowledge of CSL. The participants completed a written informed consent approved by the institutional review board of the Beijing Normal University Imaging Center for Brain Research. The consent form, safety instruction, and experimental procedures were shown in written Chinese language. A sign language interpreter helped us explain these materials and communicate with deaf signers for any questions. The characteristics of the participants are presented in **Table 1**.

**Table 1:** Background information of the 13 deaf subjects and 13 hearing subjects.

Deaf Subject	Age	Sex	Age of CSL acquisition	Residual hearing(dB)	Hearing subject	Age	Sex
D1	22	F	3	100	H1	21	F
D2	22	F	8	100	H2	20	F
D3	19	M	7	105	H3	20	F
D4	20	F	4	105	H4	21	F
D5	21	M	9	95	H5	19	F
D6	17	F	5	100	H6	21	F
D7	19	F	3	100	H7	20	M
D8	20	F	7	115	H8	20	F
D9	22	F	2	100	H9	23	M
D10	21	F	4	100	H10	19	F
D11	20	F	12	95	H11	22	F
D12	21	F	11	100	H12	20	F
D13	21	F	9	115	H13	21	F


#### Materials and Procedure

**FIGURE 1:**
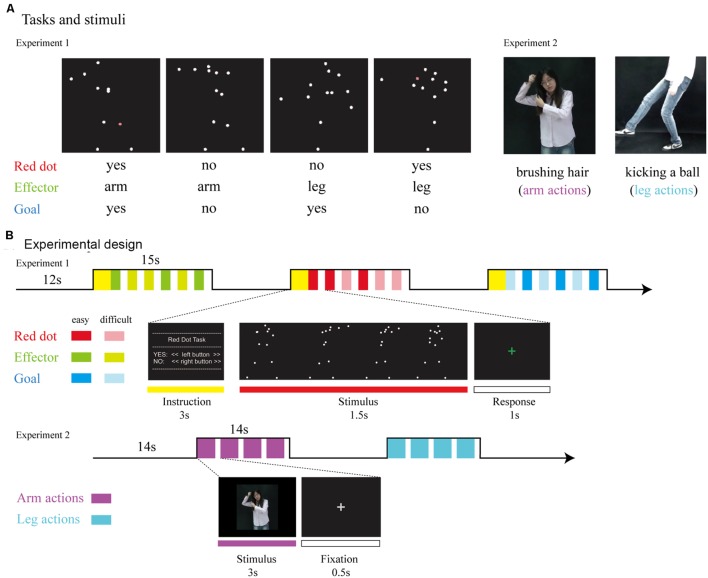
**(A)** Tasks and stimuli. For Experiment 1, stimuli consisted of point-light displays depicting four different actions (throwing a ball, punching someone, kicking a ball, and kicking someone). During the Red Dot Task, participants had to indicate whether or not 1 of the 13 markers had briefly turned red. During the Effector Task, participants had to indicate whether the relevant effector was the arm (as in throwing a ball) or the leg (as in kicking a ball). During the Goal Task, participants had to indicate whether the point-light display depicted an action involving a ball (as in throwing a ball) or not (as in punching someone). For Experiment 2, stimuli consisted of video clips which were recoded by a hearing female with no knowledge of CSL performing arm and leg actions. **(B)** Experimental design. For Experiment 1, we used a mixed design, with task blocked (15 s task, followed by 12 s rest). Within each block, the noise level was varied from trial to trial, with each noise level occurring three times per block, leading to six trials per block. The type of action was assigned randomly. For Experiment 2, stimuli were presented in 14-s blocks consisting of four video clips of either arm or leg actions.

The experimental materials and the procedure were adapted from Lingnau and Petris (2013), as presented in **Figure 1**. Briefly, point-light displays were based on digitized movements of human actors. Thirteen reflective markers were attached to the head, shoulders, elbows, hands, hips, knees, and feet. Each point-light display depicted one action lasting 1.5 s. There were four different types of human actions that involved either the hand or leg: throwing a ball, kicking a ball, punching someone and kicking someone. To manipulate task difficulty, original trajectories of six randomly selected markers were randomly rotated by 90°, 180°, or 270°, resulting in two difficulty levels (easy/original trajectories; difficult/rotated markers).

Participants responded by pressing buttons with their right index and middle fingers. For the Red Dot Task, they indicated whether any one of the 13 markers had briefly turned red. For the Effector Task, participants indicated whether the display depicted the action of the arm (throwing a ball/punching someone) or the action of the leg (kicking a ball/kicking someone). For the Goal Task, participants were required to judge whether the action involved a ball (throwing/kicking a ball) or not (punching/kicking someone). Both the Effector and Goal task required a certain degree of action understanding and was combined in our analyses as the action judgment tasks.

The experiment used a mixed design (for details, see Lingnau and Petris, 2013). Task was blocked, whereas the type of effector (arm, leg) and goal (action involving a ball or no ball) were assigned randomly within blocks. Blocks lasted 15 s and consisted of six trials. Each trial lasted 1.5 s, with a 1-s inter-trial-interval during which participants were asked to respond. There was a 3-s written instruction to inform the participants of the next task before each block and a 12-s rest afterward. Participants performed six runs. Each run lasted for 6 min 15 s and contained 12 blocks, with four blocks for each task (Red Dot, Effector, Goal) and 36 trials for each effector (arm, leg). Prior to scanning, participants were given written instructions and performed several practice blocks to ensure that they understood the requirements correctly.

Participants viewed the stimuli through a mirror attached to the head coil adjusted to allow foveal viewing of a back-projected monitor (refresh rate: 60 Hz; spatial resolution: 1024 × 768). The width and height of the point-light stimuli were approximately 14.7° × 11.1° on the screen. The size of each single dot was approximately 0.24°. ASF (Schwarzbach, 2011) based on MATLAB (MathWorks, Inc., Natick, MA, USA) and Psychtoolbox-3 (Brainard, 1997) was used to present the stimuli.

#### MRI Data Acquisition

Structural and functional MRI data were collected with a 3-T Siemens Trio Tim scanner at the Beijing Normal University MRI center. A high-resolution 3D structural data set was acquired with a 3D-MPRAGE sequence in the sagittal plane (TR: 2530 ms, TE: 3.39 ms, flip angle: 7°, matrix size: 256 × 256, 144 slices, voxel size: 1.33 × 1 × 1.33 mm, acquisition time: 8.07 min). Functional data were measured with an EPI sequence (TR: 2000 ms, TE: 30 ms, flip angle: 90, matrix size: 64 × 64, voxel size: 3.125 × 3.125 × 4 mm, inter-slice distance: 4.6 mm, number of slices: 33; slice orientation: axial).

#### Data Analysis

##### Preprocessing

Functional imaging data were analyzed using the Statistical Parametric Mapping package (SPM 12, Wellcome Department of Cognitive Neurology, London, UK). The first nine volumes were discarded. Preprocessing of functional data included head motion correction with respect to the first (remaining) volume of the run scanned closest to the 3D structural data, slice timing correction (ascending interleaved order), and spatial smoothing (Gaussian filter, 8-mm Full Width Half Maximum). For each participant, functional data were registered to her/his high-resolution structural data. Finally, both functional and structural data were normalized into Montreal Neurological Institute (MNI) space, and functional data were resampled to 3 mm × 3 mm × 3 mm resolution.

##### Whole-brain analysis

To carry out whole brain group data analysis, we used the general linear modelling (GLM), including six motion parameters as regressors of no interest. We combined the Effector and Goal tasks to a common regressor labeled ‘Action Judgment Task.’ All incorrect trials were excluded. To identify brain areas recruited while participants are engaged in the judgment of an action, we computed the contrast between the beta estimates obtained during the Action Judgment Task and the Red Dot Task. We conducted a within-group analysis and then performed a group comparison to examine the differences between deaf and hearing participants. Correction for multiple comparisons was performed using false discovery rate (FDR) correction (*q* < 0.05).

##### Regions of interest (ROI) analysis

Regions of interests were defined using the data from all of the participants (deaf and hearing individuals combined), using the contrast Action Judgment Tasks > Red Dot Task (FWE corrected, *P <* 0.05), to identify regions recruited by action understanding without biasing toward either subject groups or either action understanding tasks. We used FWE correction here because using the FDR correction threshold very large continuous clusters were obtained covering different frontal, parietal, and temporal regions. Thus the higher threshold was used to separate the continuous clusters into different clusters with different activation peaks. Beta estimates of all voxels falling within a 6 mm radius sphere centered on the peak voxel within each ROI for each factor (effector or task) of each subject group were then extracted and analyzed.

Within these ROIs, we performed three different types of repeated measures ANOVAs. (1) To examine the effect of effectors (arm, leg) on the neural activity of brain regions involved during the judgment of an action (in comparison to a low level visual control task), we carried out a repeated ANOVA on the beta estimates for the contrast ‘Action Judgment Task > Red Dot Task’ (2) To examine group differences specifically for the goal task (rather than collapsing across the effector and the goal task), we carried out a repeated measures ANOVA with the factors effector (arm, leg) and group (deaf, hearing) on the beta estimates for the contrast ‘Goal task > Red Dot Task.’ (3) To examine the effect of the two action understanding tasks (Goal Task. Effector Task), we carried out a repeated measures ANOVA with the factor task the beta estimates for the contrast ‘Goal Task > Red Dot Task’ and ‘Effector Task > Red Dot Task.’

### Results

#### Behavioral Data

Behavioral data were collected inside the MR scanner. The result of accuracy and RT are shown on **Table 2**. Accuracy and RT were analyzed using a 3 × 2 × 2 ANOVA including Task (Red Dot, Effector, Goal) and Effector (arm, leg) as within-subject factors and Group (deaf, hearing) as the between-subject factor.

**Table 2:** RT and accuracy (Experiment 1).

Task	Action type	RT (*ms*)		Accuracy (% correct)	
			
		Deaf	Hearing	Deaf	Hearing
Red dot	Arm	531.28 ± 70.7	560.59 ± 94.35	0.82 ± 0.08	0.75 ± 0.1
	Leg	498.38 ± 73.05	519.82 ± 95.83	0.87 ± 0.06	0.79 ± 0.07
Effector	Arm	500.33 ± 78.69	550.04 ± 100.65	0.92 ± 0.03	0.93 ± 0.05
	Leg	489.28 ± 63.3	539.85 ± 95.57	0.89 ± 0.06	0.86 ± 0.07
Goal	Arm	538.22 ± 71.62	576.06 ± 87.2	0.81 ± 0.07	0.77 ± 0.1
	Leg	498.18 ± 75.01	540.86 ± 97.07	0.8 ± 0.05	0.8 ± 0.05


The analysis of accuracy revealed significant main effects of Task [*F*(2,48) = 39.74, *P <* 0.001] and Group [*F*(1,24) = 4.25, *p* = 0.05] and no effect of Effector [*F*(1,24) = 0.15, *p* = 0.70]. We also found a significant interaction between Task and Group [*F*(2,48) = 3.38, *P <* 0.05] and a significant interaction between Task and Effector [*F*(2,48) = 11.90, *P <* 0.001]. During the Red Dot Task, deaf participants performed better than the hearing participants [*t*(25) = 2.63, *P <* 0.05]. No group difference was found for the other two tasks. The interaction between Effector and Group [*F*(1,24) < 0.001, *P >* 0.99] and the interaction among these three factors were not significant [*F*(2,48) = 2.37, *p* = 0.10].

For RT, there was no difference between participant groups [*F*(1,24) = 1.46, *p* = 0.24]. We found significant main effects of task [*F*(2,48) = 6.68, *P <* 0.01] and effector [*F*(1,24) = 54.93, *P <* 0.001], and a significant interaction between task and effector [*F*(2,48) = 5.74, *P <* 0.01]. RT for the Goal Task was longer than that of the Red Dot Task [*t*(25) = 2.15, *P <* 0.05], whereas the other comparisons revealed no significant difference (all *Ps >* 0.05). Longer RTs were associated for the arm than the leg in the Red Dot Task [*t*(25) = 6.29, *P <* 0.001] and Goal task [*t*(25) = 5.78, *P <* 0.001].

Taken together, there was a tendency for deaf individuals to perform better than hearing control participants in low level visual detection (Red Dot Task), but there was little evidence for behavioral differences between the two groups in the two action understanding tasks. Next we tested whether activation in the ROI was modulated by behavioral differences. To this aim we correlated behavoiral performance (RT, accuracy) with the mean beta estimate obtained from the contrast (action judgment > red dot; see below) in each ROI across participants. We found no significant correlation in any ROI for either accuracy or RT (Bonferroni corrected *P*s > 0.05).

#### Whole-Brain Analyses [Action Judgment Task > Red Dot Task; Action Judgment Task > Fixation]

**FIGURE 2:**
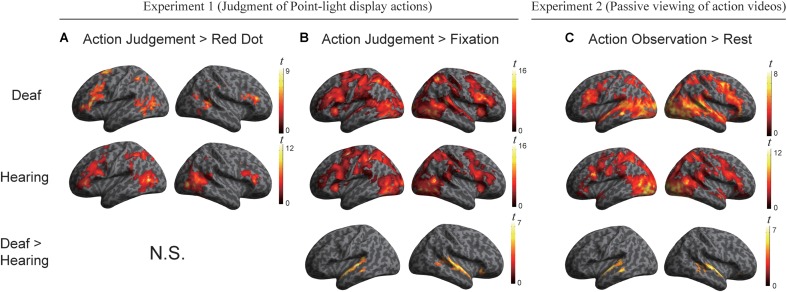
**(A)** Areas engaged in action understanding by the contrast Action Judgment Task > Red Dot Task in Experiment 1 separately for the two groups (top two rows) and comparison between groups (bottom row). There is no significant cluster revealed by the contrast Hearing > Deaf or Deaf > Hearing (FDR *q* < 0.05). **(B)** Areas revealed by the contrast Action Judgment Task > Rest (fixation baseline) in Experiment 1 separately for the two groups (top two rows) and comparison between groups (bottom row). The large cluster in the bottom row was one contiguous cluster in volume space but not when displayed on the surface. There is no significant cluster revealed by the contrast Hearing > Deaf (FDR *q* < 0.05). **(C)** Areas revealed by the contrast Action Observation > Rest (fixation baseline) in Experiment 2 separately for the two groups (top two rows) and comparison between groups (bottom row). There is no significant cluster revealed by the contrast Hearing > Deaf (FDR *q* < 0.05).

We conducted a whole-brain group comparison using the contrast Action Judgment Task > Red Dot Task (FDR *q* < 0.05). In the deaf individuals, this contrast revealed the following areas: bilateral triangular parts of IFG, the left superior parietal gyrus (SPL), the left anterior inferior parietal gyrus (aIPL), the left MOG, the pMTG, the right inferior temporal gyrus (ITG), the right superior temporal sulcus (STS), and bilateral cerebellum (**Figure 2A**, first row). In the hearing individuals, a similar pattern was obtained: bilateral triangular parts of IFG, the left pMTG, the left supplementary motor area (SMA), the right middle occipital gyrus MOG, the right IPL, the right precuneus and the bilateral cerebellum (**Figure 2A**, second row). Detailed information about the activation peak coordinates and cluster sizes is given in **Table 3**. Critically, there was no group difference between hearing and deaf individuals (FDR *q* < 0.05).

**Table 3:** Montreal Neurological Institute (MNI) coordinates of the regions revealed by the contrast Action Judgment Task > Red Dot Task for the separate group in Experiment 1.

Region	MNI coordinate			*T* value	Cluster size
			
	*x*	*y*	*z*		
**Deaf**					
L.aIPL	-42	-46	47	7.72	309
L.Cerebellum	-6	-82	-37	5.92	55
L.IFG_Tri	-48	29	14	9.96	815
L.MOG	-39	-64	5	7.61	347
L.SPL	-12	-76	53	7.37	119
R.Cerebellum	18	-73	-46	5.70	119
R.Cerebellum	24	-67	-28	4.82	33
R.Cerebellum	12	-85	-31	4.78	22
R.IFG_Tri	45	32	17	7.54	192
R.ITG	48	-61	-4	4.47	24
R.pMTG	45	-61	23	4.52	26
R.pSTS	54	-43	8	8.47	147
**Hearing**					
L.Cerebellum	-9	-85	-34	8.31	223
L.IFG_Tri	-48	38	5	10.05	1165
L.pMTG	-48	-67	8	13.43	1496
L.SMA	-6	20	47	5.70	102
R.aIPL	45	-34	50	5.92	58
R.Cerebellum	21	-76	-40	8.69	411
R.IFG_Tri	48	35	11	8.07	417
R.MOG	45	-76	5	9.98	1029
R.Precuneus	21	-58	26	4.71	27


*The false positive rate was set at *q* < 0.05 corrected with false discovery rate (FDR).*

We further carried out another contrast using a less specific contrast: Action Judgment Task > Rest (Fixation) corrected at FDR *q* < 0.05. This contrast results in cognitive components of action understanding, as well as low level visual processes. When comparing hearing and deaf individuals, we found stronger activation in deaf in comparison to hearing individuals in very large clusters around the bilateral auditory cortex: the left cluster encompassed almost the entire STG, which extended to the pMTG [peak, -60, -22, 2; size, 373 voxels]; the right cluster included the right STG, extending to pMTG and orbital IFG [peak, 60, -1, -7, size, 659 voxels]; see **Figure 2B**, bottom row, in which the large clusters was one contiguous cluster in volume space but not when displayed on the surface.

#### ROI Analyses Testing the Effects of Effector, Task, and Deafness

**FIGURE 3:**
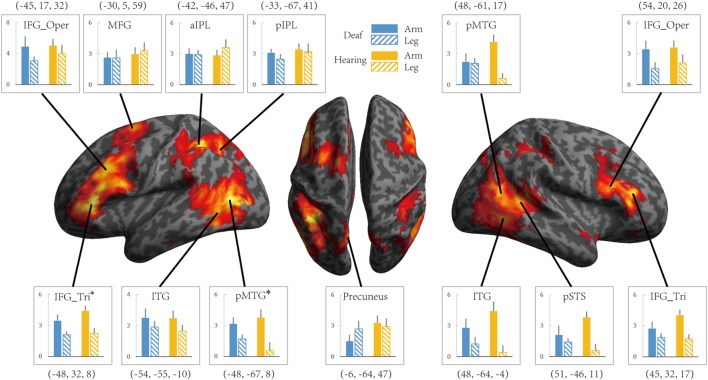
**Areas recruited in action understanding (contrast Action Judgment Task > Red Dot Task) in Experiment 1 for all participants (FDR *q* < 0.05).** ROIs were defined using the same contrast but at a stricter threshold (FWE *P* < 0.05). The blue and yellow bars depict beta estimates in the hearing and deaf group, respectively. Solid and slash bars represent the arm and leg actions, respectively. Asterisk indicates significant main effect between arm and leg condition. (*P* < 0.004, significant at Bonferroni corrected 0.05 for multiple comparison across 13 ROIs).

To investigate whether the effects of group (deaf vs. hearing) on activation during action understanding is modulated by effector (arm, leg), task (Effector Task, Goal Task), or sign language experience, we conducted a series of ROI analysis in areas recruited during the action judgment tasks. Following Lingnau and Petris (2013), we defined ROIs by the GLM contrast Action Judgment Task (Goal + Effector Task) > Red Dot Task (FWE corrected, *P* < 0.05). We collapsed across the two groups and the two tasks in order to avoid biased ROI selection (Kriegeskorte et al., 2009). This contrast revealed 13 ROIs: bilateral ITG, bilateral triangular and opercular parts of IFG, bilateral posterior MTG, left anterior and posterior IPL, left middle frontal gyrus (MFG), left precuneus and right posterior STS (**Figure 3**). The ROIs opercular part of IFG, the IPL, and the pSTS were within the traditional hMS regions. Beta estimates revealed by this contrast are shown in **Figure 3**, separately for each ROI.

Whether the potential difference between deaf and hearing groups is modulated by action effector type was examined by the repeated-measures ANOVA with the factors Group and Effector, the results of which are presented in **Table 4** for each ROI. For the left triangular part of IFG and the pMTG, the main effect of Effector was significant (*P* < 0.004, Bonferroni corrected *P* < 0.05), with both regions showing a higher BOLD response for arm actions than leg actions in all of the ROIs. Importantly, no regions showed a main effect of Group or an interaction between Effector and Group.

**Table 4:** Statistical details for main effect of Effector, Group, Interaction Effector × Group and R value with CSL acquisition age with Deaf individuals by the contrast Action Judgment Task > Red Dot Task in Experiment 1 (^∗^*P* < 0.004, significant at Bonferroni corrected 0.05 for multiple comparisons across 13 ROIs).

	Main effect of effector		Main effect of group		Interaction Effector × Group		R value with CSL acquisition age with deaf individuals	
				
	*F*(1,23)	*P*	*F*(1,23)	*P*	*F*(1,23)	*P*	*R*	*P*
L.aIPL	0.42	0.52	0.2	0.66	0.63	0.44	0.01	0.99
L.IFG_Oper	2.37	0.14	0.19	0.67	0.19	0.67	0.23	0.47
L.IFG_Tri	13.951	<0.01^∗^	1.03	0.32	0.72	0.41	0.49	0.11
L.ITG	3.21	0.09	0.05	0.82	0.09	0.77	0.31	0.33
L.MFG	0.05	0.83	0.53	0.48	0.06	0.81	0.19	0.55
L.pIPL	0.6	0.45	0.71	0.41	0.11	0.74	0.22	0.49
L.pMTG	11.39	<0.01^∗^	0.11	0.74	1.52	0.23	-0.25	0.44
L.Precuneus	0.31	0.58	1.69	0.21	1	0.33	0.23	0.47
R.IFG_Oper	5.97	0.02	0.17	0.68	0.07	0.8	0.32	0.32
R.IFG_Tri	7.84	0.01	1.09	0.31	1.68	0.21	0.44	0.16
R.ITG	9.13	0.01	0.29	0.6	1.78	0.2	0.14	0.66
R.pMTG	8.74	0.01	0.1	0.75	7.52	0.01	-0.16	0.61
R.pSTS	6.96	0.02	0.54	0.47	2.85	0.11	-0.17	0.6


It might be possible to perform the Effector Task without understanding the actions. We thus examined if there are any group differences when focusing on the contrast ‘Goal task > Red Dot Task’ (see **Table 5**). For the left pMTG, right ITG and pSTS, the main effect of Effector was significant (*P* < 0.004, Bonferroni corrected at *P* < 0.05), with both regions showing a higher BOLD response for leg actions in comparison to arm actions. No regions showed a main effect of Group or an interaction between Effector and Group.

**Table 5:** Statistical details for main effect of Tasks (Goal task/Effector task), Group, Interaction Tasks × Group and R value in Experiment 1 (^∗^*P* < 0.004, significant at Bonferroni corrected 0.05 for multiple comparisons across 13 ROIs).

	Main effect of tasks		Main effect of group		Interaction Tasks × Group	
			
	*F*(1,19)	*P*	*F*(1,19)	*P*	*F*(1,19)	*P*
L.aIPL	7.58	0.01	0.11	0.74	0.09	0.76
L.IFG_Oper	34.16	<0.01^∗^	0.02	0.90	0.55	0.46
L.IFG_Tri	52.17	<0.01^∗^	4.64	0.04	0.00	0.98
L.ITG	0.93	0.35	6.78	0.02	0.18	0.67
L.MFG	10.70	<0.01^∗^	1.24	0.28	0.00	0.99
L.pIPL	0.84	0.37	6.12	0.02	0.22	0.64
L.pMTG	0.34	0.57	0.08	0.78	1.40	0.25
L.Precuneus	2.74	0.11	0.03	0.87	1.68	0.21
R.IFG_Oper	24.38	<0.01^∗^	9.48	0.01	0.15	0.70
R.IFG_Tri	40.66	<0.01^∗^	6.75	0.02	0.02	0.88
R.ITG	1.27	0.27	3.02	0.10	2.04	0.17
R.pMTG	1.06	0.31	0.00	0.97	0.02	0.90
R.pSTS	2.60	0.12	9.97	0.00	0.26	0.62


We further tested whether the group difference is affected by the type of task using a repeated measures ANOVA with the factors Group (deaf vs. hearing) and Task (Goal vs. Effector Task; see **Table 5**). For the left triangular part and opercular part of IFG and the MFG, right triangular part and opercular part of IFG, the main effect of Task was significant (*P* < 0.004, Bonferroni corrected *P* < 0.05), with a higher BOLD amplitude for the Goal Task in comparison to the Effector Task. Importantly, however, no region showed a main effect of Group or an interaction between Task and Group.

#### Testing the Effects of Sign Language Experience within the Deaf Group

To test whether activation in ROIs identified by the contrast Action Judgment Task > Red Dot Task is modulated by sign language experience, we computed the correlation between the beta value of each ROI and the age of CSL acquisition, a common measure of language experience, across deaf subjects. We obtained no significant correlation in any ROI (*P*s > 0.1, **Table 4**).

We further divided the deaf subjects into two groups according to the age of CSL acquisition (>6 years, *N* = 6 vs. <6 years, *N* = 6, results presented in **Table 6**). Two sample *t*-test was used to test whether the two groups differed in each ROI. No significant differences were obtained in any ROI (Bonferroni corrected *Ps* > 0.05).

**Table 6:** Statistical details for comparing two sub-groups of deaf individuals (the age of acquisition CSL > 6 years vs. < 6 years) in Experiments 1 and 2 (*P* > 0.004, no significant result at Bonferroni corrected.05 for multiple comparisons across 13 ROIs).

	Experiment 1				Experiment 2			
		
	Arm		Leg		Arm		Leg	
				
	*t*(10)	*P*	*t*(10)	*P*	*t*(8)	*P*	*t(8)*	*P*
L.aIPL	0.12	0.91	0.36	0.73	-1.05	0.32	-1.73	0.12
L.IFG_Oper	1.46	0.18	1.09	0.30	-0.38	0.72	-0.34	0.74
L.IFG_Tri	-0.66	0.53	2.74	0.02	-0.47	0.65	0.51	0.62
L.ITG	0.75	0.47	1.21	0.25	0.37	0.72	1.23	0.25
L.MFG	1.61	0.14	1.02	0.33	-0.67	0.52	-0.56	0.59
L.pIPL	-0.08	0.94	0.80	0.44	-0.11	0.92	-3.25	0.01
L.pMTG	-1.40	0.19	-0.85	0.41	1.39	0.20	1.36	0.21
L.Precuneus	0.49	0.63	1.04	0.32	-0.02	0.99	-1.31	0.23
R.IFG_Oper	0.76	0.46	1.12	0.29	1.00	0.35	1.19	0.27
R.IFG_Tri	1.19	0.26	1.70	0.12	0.46	0.66	-0.02	0.99
R.ITG	-0.18	0.86	0.37	0.72	1.01	0.34	1.34	0.22
R.pMTG	-1.07	0.31	-1.08	0.31	0.16	0.87	0.34	0.74
R.pSTS	0.34	0.74	-0.10	0.92	0.60	0.57	1.02	0.34


## Experiment 2: Passive Viewing of Action Videos

### Methods

#### Participants

Ten deaf and 11 hearing individuals from Experiment 1 took part in Experiment 2.

#### Materials and Procedure

To be as close as possible to previous studies on action observation in deaf participants (Corina et al., 2007; Emmorey et al., 2010), we used action videos instead of point light displays in Experiment 2 (see **Figure 1**). A hearing female with no knowledge of CSL performed arm and leg actions that were recorded with a video camera and edited into video clips. The arms actions consisted of the hand manipulating a variety of common objects (objects not presented) or acting upon a part of the body, such as brushing hair, knocking at the door and washing hands. Leg actions were performed by the leg and foot, such as walking, running and kicking a ball. In total there were 48 arms action video clips and 48 arm action clips, created from 28 different arm actions and 24 leg actions (see the full list of materials in Appendix A) by repeating each action twice or occasionally once.

In the scanner, stimuli were presented in 14-s blocks consisting of four video clips of either arm or leg actions. There were two runs, each consisted of 12 blocks, with six blocks for each type of effector, and lasted for 5 min 50 s. Within each run, the order of blocks was randomized and each action appeared once. Within a block, each video clip lasted for 3 s, followed by a 500-ms fixation cross. Between blocks, as well as before the first and after the last block, a baseline condition, consisting of a fixation cross, was presented for 14 s. The width and height of the video clips were approximately 16.8° × 12.6° on the screen. Psychtoolbox-3 was used for controlling stimulus presentation.

Participants performed a passive viewing task. Following Emmorey et al. (2010), participants were instructed to pay attention to all videos without explicitly trying to memorize them.

After scanning, participants received a short recognition test to examine whether they observed the stimuli in the scanner. Twenty-five video clips were presented, of which five clips were novel, and 20 clips were shown in the scanner. The participants were asked to judge whether they had seen them during the experiment. The mean accuracy was 78% for the deaf group and 70% for the hearing group, with no significant difference between groups [*t*(19) = 0.96, *P >* 0.05].

#### MRI Data Acquisition, Preprocessing, and Analyses

An identical procedure to Experiment 1 was used for data collection, preprocessing, and whole brain analysis of the fMRI data. The only exceptions were that the first 14 s (7 volumes) in each functional run were discarded and that we did not apply slice timing correction. The critical contrast was the comparison between the action observation condition and the fixation baseline. In parallel to Experiment 1, we carried out whole brain analysis and ROI analysis to test whether the deaf and hearing groups differed in terms of brain activation patterns when viewing actions.

### Results

#### Whole-Brain Analyses [Action Observation > Fixation]

**FIGURE 4:**
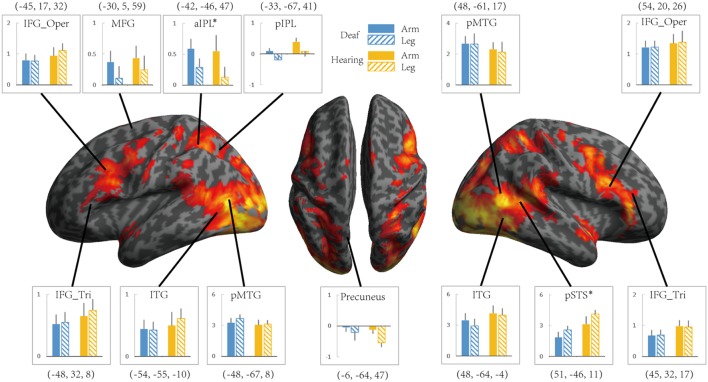
**The effect of the Effector for Action Observation in Experiment 2.** Areas recruited during action observation (contrast Action Observation > Fixation baseline) in Experiment 2 for all participants (FDR *q* < 0.05). ROIs were defined in Experiment 1 by the contrast Action Judgment Task > Red Dot (FWE *P* < 0.05). Colors and symbols are the same as in **Figure 3**.

Areas recruited during passive action observation were identified by the contrast Action Observation Task > Fixation (FDR *q* < 0.05), revealing widely distributed regions in the inferior frontal, parietal, posterior temporal and occipital cortex in both deaf and hearing individuals (**Figures 2** and **4**, first two rows).

The whole-brain group comparison between the two groups yielded no region that was more active for the hearing compared to deaf individuals (FDR *q* < 0.05). Regions in the bilateral superior and middle temporal gyrus showed significantly stronger activation in deaf individuals than in hearing controls [for left, peak, -63, -10, -1, size, 295 voxels; for right, peak, 63, -10, 2, size, 344 voxels] (**Figure 2**, bottom row).

#### ROI Analyses Testing the Relationship between Group and Effector

We further tested the effects of Group and Effector in the ROIs identified in Experiment 1 (see **Figure 3** for the ROIs) by carrying out ANOVA of 2 (deaf vs. hearing) × 2 (arm vs. leg) for each of the ROIs, with the dependent variable being the beta estimate of the contrast (action passive viewing vs. fixation). The result is shown in **Figure 4** and **Table 7**. We found a main effect of Effector in two regions: left aIPL [*F*(1,19) = 12.97, *P <* 0.005] and right pSTS [*F*(1,19) = 26.13, *P <* 0.001]. Arm actions evoked a greater activation than leg actions in left aIPL, whereas we found the opposite pattern in the right pSTS. No region showed the main effector of Group or an interaction between Effector and Group.

**Table 7:** Statistical details for main effect of Effector, Group, Interaction Effector × Group and R value with CSL acquisition age with deaf individuals during action observation in Experiment 2 (asterisk indicates *P* < 0.004, significant at Bonferroni corrected 0.05 for multiple comparisons across 13 ROIs).

	Main effect of effector		Main effect of group		Interaction Effector × Group		*R* value with CSL acquisition age with deaf individuals	
				
	*F*(1,19)	*P*	*F*(1,19)	*P*	*F*(1,19)	*P*	*R*	*P*
L.aIPL	12.97	<0.01^∗^	0.12	0.73	0.38	0.54	0.39	0.27
L.IFG_Oper	0.28	0.6	0.56	0.46	0.6	0.45	0.10	0.78
L.IFG_Tri	0.49	0.49	0.4	0.53	0.13	0.73	0.11	0.76
L.ITG	0.25	0.62	0.27	0.61	0.49	0.49	-0.29	0.42
L.MFG	2.67	0.12	0.14	0.71	0.07	0.79	0.31	0.38
L.pIPL	6.9	0.02	2.64	0.12	0.02	0.89	0.54	0.11
L.pMTG	3.91	0.06	0.32	0.58	1.69	0.21	-0.19	0.60
L.Precuneus	3.88	0.06	0.87	0.36	0.82	0.38	0.10	0.79
R.IFG_Oper	0.02	0.88	0.13	0.73	<0.01	0.95	-0.36	0.31
R.IFG_Tri	0.01	0.93	1.07	0.31	0.04	0.85	-0.13	0.72
R.ITG	5.16	0.04	0.87	0.36	1.21	0.29	-0.28	0.43
R.pMTG	0.44	0.52	0.25	0.62	0.35	0.56	0.02	0.96
R.pSTS	26.13	<0.01 ^∗^	3.54	0.08	0.51	0.48	-0.12	0.73


#### Testing the Effects of Sign Language Experience within the Deaf Group

For the ROIs described above, no region exhibited a significant association between the strength of the BOLD signal and the age of CSL acquisition across deaf participants (*P*s > 0.1, **Table 7**). When the deaf individuals were divided into groups with early or late CSL acquisition (see Experiment 1, **Table 6**), no difference was observed between the two groups in any ROI (Bonferroni corrected *P* < 0.05).

## Discussion

In this study, we used both an action judgment task and a passive action observation task to investigate whether the processing of arm- and leg-related actions is affected by auditory experience deprivation and/or sign language experience. We found highly similar activation in the action observation network in congenitally deaf and hearing individuals in both action judgment and passive action observation tasks. Whole brain analyses showed that both groups recruited a comparable network of parieto-frontal regions, including IFG, IPL, and pMTG. Both the whole brain analyses and the ROI analyses showed that the activation amplitude between the two groups were comparable. Likewise, the potential effects of effector type (arm, leg) or task (Effector task, Goal task) did not differ across groups, and that the activation in the action observation network within the deaf group did not seem to be influenced by sign language experience. Taken together, we observed that the action-understanding-related areas were similarly recruited when congenitally deaf participants or hearing controls processed action stimuli, in both action understanding tasks and a passive viewing task.

We aimed at evaluating the origins of the absence of hMS activation during action observation previously reported in congenitally deaf individuals by assessing the effects of task, effector type, and sign language experience. In contrast to previous studies (Corina et al., 2007; Emmorey et al., 2010), we observed robust activation in IFG and IPL in both hearing and deaf individuals. In addition, both groups showed robust activation in STS and pMTG, highly consistent with more recent studies on the neural basis of action understanding (Oosterhof et al., 2012; Lingnau and Petris, 2013; Lingnau and Downing, 2015; Tarhan et al., 2015; Wurm and Lingnau, 2015; Wurm et al., 2015). That is, both the hMS (IFG, IPL, STS) and pMTG are resilient to the auditory experience deprivation and to the adoption of a new action system, i.e., sign language. While both hemispheres were activated, there was a tendency of left lateralization, with larger regions in the left hemispheric (left MFG and left IPL) being recruited during action understanding in both groups, in line with the literature on hearing populations (Rocca et al., 2008; Lingnau and Petris, 2013).

Note that ideally adding a hearing sign user group and/or a deaf group without sign language experience would help tease apart the roles of auditory experience and sign language experience. Also note that we here tested the effects of sign language experience rather than sign language fluency. It would be interesting to further examine whether there is subtle modulation effect of hMS activation associated with fluency. However, given that we observed comparable-to-hearing hMS activation in congenital deaf groups who use CSL as the primary language, it is safe to conclude that hMS activation is robust to the presence of both auditory deprivation and sign language usage. Below we discuss possible reasons for the discrepancy between our results and the two previous studies showing no hMS activation in deaf (Corina et al., 2007; Emmorey et al., 2010), in the context of implications of these results in the functional properties of areas involved during action understanding.

Several aspects differing between our current study and the previous studies are worth considering, including tasks, stimulus properties, and subject characteristics. In Experiment 1 we used point-light animations and tasks that require understanding, as opposed to the passive viewing task used in the previous two studies where understanding was not necessary. In Experiment 2, we administered a passive viewing task similar to that used by Corina et al. (2007) and Emmorey et al. (2007), but found similar results as in Experiment 1. That is, the mismatch between our results and those observed by Corina et al. (2007) and Emmorey et al. (2010) cannot readily be explained by task variables.

The difference in subject sampling might be more relevant. Deaf individuals in the previous two studies were all native signers who were exposed to ASL from birth, whereas in our cohort only three individuals were native signers. Emmorey et al. (2010) contended that sign language experience improved the neural efficiency within the hMS, leading to reduced activation. However, not only that this proposal does not explain the absence of activation, it was further challenged by several additional observations. First, we did not obtain any correlation between sign language experience and activation strength in the hMS in either experiment. Second, given that CSL primarily involves hand and arm action, if CSL experience modulates hMS activation, one would predict that hand/arm and leg actions are processed differently in CSL users and non-users. However, while observing main effects of the effector in the hMS and other action related regions, we observed no interaction with the subject group. That is, arm actions were not treated differently by signers and by hearings subjects. Finally, we carried out individual analyses with the three native signers in our deaf group and observed robust activation of the hMS (IFG, IPL) and pMTG in each of these individuals.

Another intriguing difference lies in the properties of the stimuli. We specifically focused on avoiding actions that resembled sign language symbols in Experiment 2, which aimed to understand the general processing mechanism of actions when deaf participants viewed passive action video clips following previous studies. To assess whether the video stimuli contain the CNL symbols, we asked two native CNL deaf signers and a hearing sign language interpreter to judge whether the video stimuli were CNL symbols. All video stimuli were judged as “not CNL symbols.” While in both Corina et al. (2007) and Emmorey et al. (2007) ASL conditions were included to compare the activation in the hMS with and without linguistic processing. It is possible that processing sign language entails linguistic properties such as lexical, semantic, “phonological” or syntactic processing that are also processed by (subregions of) the hMS, resulting in the different hMS activations. Although most stimuli in Emmorey et al. (2010) were judged not to be linguistic, it is conceivable that the inclusion of even a small proportion of linguistically meaningful actions may encourage linguistic interpretation of the non-linguistic actions and thus affect the hMS activation. Note, however, that Corina et al. (2007) and Emmorey et al. (2007) did not obtain consistent results regarding sign language processing: while Corina et al. (2007) found a marked difference between the brain regions subserving linguistic and non-linguistic human actions in deaf signers, in the study of Emmorey et al. (2007), no region was significantly more engaged in processing ASL verbs compared to pantomimes. Emmorey et al. (2007) suggested that the pantomimes they used contained sentence-level concepts with both an agent and a patient, which evoked more extensive semantic processing than the ASL condition. The roles of the hMS and other action-related regions in sign language action processing warrant further clarifications (Neville et al., 1998; Alaerts et al., 2011; Rogalsky et al., 2013).

A few other findings outside the hMS are worth further discussion. First, when contrasting with fixation, regions outside of the action understanding network were observed, mostly in the primary visual cortex, likely reflecting responses to the presence of (low level) visual stimulation. More interestingly, using this contrast the deaf individuals showed stronger auditory cortex activation to visual stimuli (in comparison to the fixation baseline) than hearing controls. This is well in line with the classical literature of plastic changes in the auditory cortex in the case of auditory deprivation (Finney et al., 2001, 2003; Pekkola et al., 2005). Another finding is more puzzling. The regions showing significant effector effects differed across the two experiments, with left IFG and pMTG showing stronger responses to arm actions than leg actions in the action judgment task, and the left IPL and right pSTS showing stronger responses to arm and leg actions respectively in the passive viewing experiment. This difference might be related to the different arm and action stimuli used in the two experiments: Experiment 1 used simple throwing and kicking action point light displays with minimal object implications, while Experiment 2 used action videos containing highly rich contents implying various specific objects such as peeling a banana, playing a piano, or dancing a waltz. Such implied information associated with the specific actions may further modulate the hMS regions in complex manners. The important point in the current context, however, is that in neither experiment the effector effect interacted with groups, indicating that whatever variables caused the differences of effectors, they were not modulated by auditory experience.

Our results, that the action observation network is similarly recruited in individuals without hearing experience and with rich sign language experiences, corroborate previous studies demonstrating the recruitment of IFG, IPL, and pMTG during the recognition of sounds depicting actions in congenitally blind individuals (Ricciardi et al., 2009; Lewis et al., 2011). These results together suggest that some of the areas recruited during action understanding are not modulated by specific visual or auditory inputs. Such areas might represent or process “supra-modal” (Ricciardi et al., 2014) action knowledge that is not specific to individual modalities.

## Author Contributions

YB, ZH, and QC designed research; YF and QC performed research; YF and QC analyzed data; YF, QC, AL, and YB wrote the paper. AL provided the experimental materials.

## Conflict of Interest Statement

The authors declare that the research was conducted in the absence of any commercial or financial relationships that could be construed as a potential conflict of interest.
