# Supplementary material for: Areas Recruited during Action Understanding Are Not Modulated by Auditory or Sign Language Experience
Source: Front Hum Neurosci. 2016 Mar 8;10:94. doi: 10.3389/fnhum.2016.00094 (PMC4781852; doi:10.3389/fnhum.2016.00094)
Supplement: Supplementary file 1 [file Data_Sheet_1.DOCX]

Appendix A. Stimuli used in Experiment 2 (Passive viewing of actions)

| Arm actions | Leg actions |
| --- | --- |
| brushing hair | crouching |
| brushing teeth | dancing a waltz |
| clapping hands | doing goose step |
| giving a salute | high jump |
| grasping something to eat | jumping with one foot |
| hammering a nail | kicking a ball |
| ironing clothes | kicking a shuttlecock |
| knocking at the door | kicking someone |
| listening on the telephone | kneeling on one knee |
| opening a door | long jump |
| paintings a wall | marking time |
| parting curtains | rolling circles |
| paying a new year call | running |
| peeling a banana | sitting down |
| playing the piano | sitting up |
| pointing with fingers | sitting with legs crossed |
| putting on eyeglasses | stamping the ground |
| raising hands | standing at ease |
| screwing a cap | standing on tiptoe |
| sewing with needle and thread | taking a step back |
| shaking hands with somebody | tap dancing |
| shooting arrows | turning to the left |
| sneezing | turning to the right |
| striking a match | walking |
| tearing a sheet of paper |  |
| turning over pages |  |
| washing face |  |
| washing hands |  |
